# Little reason to call them small noncoding RNAs

**DOI:** 10.3389/fmicb.2023.1191166

**Published:** 2023-06-29

**Authors:** Silvia Ferrara, Tarcisio Brignoli, Giovanni Bertoni

**Affiliations:** Department of Biosciences, Università degli Studi di Milano, Milan, Italy

**Keywords:** small RNAs, RNA-binding proteins, bacteria, riboregulation, dual-function sRNAs, Hfq, CsrA/RsmA, small protein

## Abstract

Hundreds of different species of small RNAs can populate a bacterial cell. This small transcriptome contains important information for the adaptation of cellular physiology to environmental changes. Underlying cellular networks involving small RNAs are RNA–RNA and RNA-protein interactions, which are often intertwined. In addition, small RNAs can function as mRNAs. In general, small RNAs are referred to as noncoding because very few are known to contain translated open reading frames. In this article, we intend to highlight that the number of small RNAs that fall within the set of translated RNAs is bound to increase. In addition, we aim to emphasize that the dynamics of the small transcriptome involve different functional codes, not just the genetic code. Therefore, since the role of small RNAs is always code-driven, we believe that there is little reason to continue calling them small noncoding RNAs.

## Introduction

Recently, the explosion of metatranscriptomic analysis of complex, often non-culturable, microbial populations sampled in diverse environments has revealed a universe of unassigned sequencing reads amounting to about 50% of total non-ribosomal RNAs. This large fraction of RNAs may consist of mRNAs coding for unknown proteins, RNA regulatory elements, or RNA viruses. It was predicted that a significant portion (about 20%–30%) of these orphan RNA sequences could be assigned to small RNA (sRNA) families (Shi et al., 2009; Gosalbes et al., 2011; Gelsinger et al., 2020), where small is conventionally defined as between 50 and 300 (for some authors up to 500) nucleotides in length. Several studies on model bacteria have shown that sRNAs play a key role in gene regulatory networks involved in the physiological response to environmental changes (Caldelari et al., 2013; Wagner and Romby, 2015; Dutta and Srivastava, 2018; Felden and Augagneur, 2021). Given the predicted abundance of sRNAs expressed by environmental microbial populations, we can speculate that the regulatory role characterized in the laboratory is also critical in the environmental context.

By base-pairing, sRNAs can influence the translatability and/or stability of target mRNAs. sRNAs can also interplay with RNA-binding proteins (RBPs), modulating, e.g., by sequestration or acting as guides, their regulatory activity. Reciprocally, RBPs can influence sRNA stability and expression. The regulatory activity of sRNAs can also be challenged by other RNAs, the so-called RNA sponges, which allow the coordinated expression of different target genes. Therefore, small RNAs have several modes of action for regulating gene expression (Nitzan et al., 2017; Dutta and Srivastava, 2018; Felden and Augagneur, 2021). Because of this obvious aptitude for riboregulation and not for carrying a message for protein translation, they are often called small noncoding RNAs. However, for about ten sRNAs characterized for their riboregulatory activity through base-pairing, it was pointed out that they can also serve as mRNAs. These sRNAs form a special class of sRNAs called dual-function sRNAs, i.e., riboregulators and mRNAs (Gimpel and Brantl, 2017; Raina et al., 2018). In very few cases, sRNA duality can be at the level of regulatory activity, which can occur both by base-pairing with target mRNAs and by protein titration (Jørgensen et al., 2013).

In the past few years, protocols based on RNA-seq have revolutionized our approach to studying the transcriptome dynamics of prokaryotes (Hör et al., 2018). Referring to bacteria where they have been extensively appreciated, sRNAs can originate from the primary transcription of diverse genomic regions, both intergenic and intragenic, and also from precursors belonging to every type of RNA molecule (e.g., tRNAs, rRNAs, 5′ and 3’ UTRs of mRNAs) (Carrier et al., 2018; Adams and Storz, 2021). Therefore, the small transcriptome is very complex, with hundreds of different species of sRNAs populating a bacterial cell (Adams and Storz, 2021). Several studies indicated a similar level of sRNA complexity in archaea (Gelsinger and Diruggiero, 2018). Therefore, the RNA-seq approach has been a major contributor to the enrichment of small transcriptome datasets with several new hits in many prokaryotic species. To prevent this from remaining a mere collecting activity, we need to ask what role the sRNA members are playing. Are they mainly riboregulators with rare protein-coding members? This question could also be asked another way: to what extent does the small transcriptome overlap with the translatome? Where translatome refers to the sum of RNA sequences which are translated into proteins by the ribosomal machinery (Ruiz-Orera and Albà, 2019). Finally, the next question to be answered is how many of the sRNAs in the translatome are dual-function sRNAs or simply small mRNAs (smRNAs) that encode only small proteins and are incapable of base-pairing or interacting with RBPs, i.e., are not riboregulators.

In this perspective paper, we aim to highlight the complexity of small transcriptome functional codes that are emerging from recent approaches and the challenges of integrating their results to provide a more detailed description of the cellular networks in which sRNAs are involved.

## The small transcriptome uses a variety of functional codes

In the last few years, high-resolution transcriptome mapping obtained with different techniques based on RNA-seq, both in several longstanding and emerging model bacteria, has revealed the presence of many species of sRNAs (Hör et al., 2018; Ryan et al., 2020; Adams and Storz, 2021) other than 6S and CRISPR RNAs (Cavanagh and Wassarman, 2014; Barrangou and Horvath, 2017). It is assumed that this myriad of sRNAs acts primarily as effectors in regulatory networks of gene expression. Behind the regulatory mode of action of sRNAs, there are at least two types of interactions that can also act in concert. One is to perform RNA–RNA base-pairing, even on a limited scale, with target mRNAs and, in addition, with other sRNAs, as is the case with sponge RNAs (Denham, 2020). The other is to interact with RBPs that can act *per se* as global translation regulators by direct binding to mRNAs.

**Figure 1:**
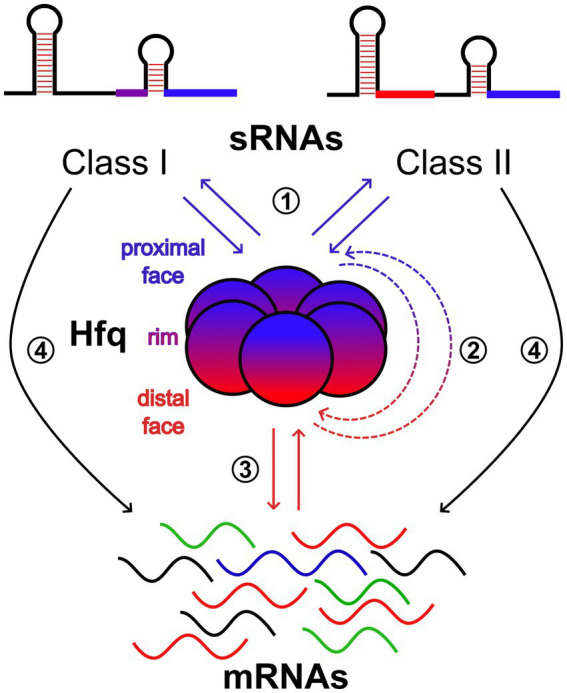
Scheme of the network of molecular interactions that take place on the faces of Hfq. The proximal and distal faces and the rim of the ring-shaped homohexamer of Hfq are shown in blue, red, and purple, respectively. For sRNAs, elements in blue represent the U-rich sequences of the intrinsic terminators used by both Class I and II sRNAs to bind to the proximal face of Hfq. Class I and II also use UA-rich motifs (purple) and A-rich motifs (red) to bind to the rim and distal face of Hfq, respectively. mRNAs containing A-rich motifs can interact with the distal face of Hfq. Those with UA-rich motifs can interact with the rim (not shown). Therefore, Class I and II sRNAs compete for the proximal face (1). Class II sRNAs, once they have gained access to the proximal face, compete with mRNAs for the distal face and vice versa (2). mRNAs compete for the distal face (3). Finally, Hfq can act as an RNA matchmaker promoting base-pairing between sRNAs and target mRNAs (4).

Many studies in the well-characterized *Escherichia coli* and *Salmonella enterica*, as well as in a broad range of bacterial species, suggest that the prevalent mode of sRNA regulatory action is through base-pairing, either negatively or positively regulating the translation and/or stability of multiple *trans*-encoded mRNAs (Adams and Storz, 2021). Although not essential, Hfq acts to facilitate base-pairing by increasing the sRNA-mRNA annealing rate in many cases (Updegrove et al., 2016; Woodson et al., 2018). Therefore, this can be considered a case where base-pairing and protein-binding activities of sRNAs work in concert for regulatory purposes. However, in addition to the role of “RNA matchmaker” (Santiago-Frangos and Woodson, 2018), there is growing evidence that Hfq is an important hub for sRNA-mediated gene regulation (Ng Kwan Lim et al., 2021). The key for this is the homo-hexameric structure of Hfq (Figure 1) that has multiple RNA-interacting interfaces, referred to as the “proximal face”, the “distal face”, and the “rim” which is the outer ring (Santiago-Frangos and Woodson, 2018). The proximal face preferably binds U-rich sequences, while the distal face prefers A-rich motifs whose compositions can be species-specific. The rim can interact with UA-rich motifs. Moreover, the Hfq unstructured C-terminal end can interact with and promote the exchange of certain RNAs. A refined model describing the complex scenario of having multiple interactions between Hfq and sRNAs/mRNAs has been provided in *E. coli* (Schu et al., 2015) and suggested the sorting of sRNAs into two classes, Class I and II (Figure 1). The Hfq proximal face is thought to be generally important for the binding of both sRNA classes through their poly-U tail of the Rho-independent terminator. Then, Class I and Class II sRNAs use the rim and distal face, respectively, as the second binding site. The preferred target mRNAs of the two sRNA classes are suggested to have complementary binding sites on Hfq. Class I and Class II sRNA-targeted mRNAs bind to the distal face and the rim, respectively, to efficiently form sRNA–mRNA complexes. Moreover, sRNA–mRNA base-pairing involves also the refolding of RNA substrates and their proper orientation at nearby sites on the rim of Hfq (Santiago-Frangos and Woodson, 2018). The positions of the A- and U-rich Hfq binding motifs, as well as the size and secondary structure, determine how a given RNA folds around the Hfq hexamer and thus influence the architectures of Hfq-RNA complexes (Santiago-Frangos and Woodson, 2018). This may provide an additional layer of specificity for sRNA-mRNA recognition. Finally, the specific orientation of the RNA on Hfq might also be important for interactions with the degradosome or other RBPs (Santiago-Frangos and Woodson, 2018).

Taken together, all these aspects suggest the presence of a multifaceted “RNA interacting code” that governs the Hfq hub and makes it very versatile and capable of multiple intertwined switches. The functional interaction of a significant portion of the small transcriptome with Hfq is a good example of the need to better understand the mechanisms behind the regulatory roles of sRNAs.

Two recent pieces of work in *E. coli* have begun to unravel this complexity. By considering only the Hfq function of RNA matchmaker, Faigenbaum-Romm et al. (2020) were able to distinguish two subsets of mRNAs, those that actively compete for association with Hfq and thus undergo strong sRNA-mediated regulation, and mRNAs that bind inefficiently to Hfq and show weak or undetectable sRNA-dependent regulation due to rare co-association on Hfq with cognate sRNA regulators. Therefore, a hierarchy of Hfq occupancy of sRNA-targeted mRNAs seems to play a major role in their regulation. But what about competition for Hfq binding among sRNAs within classes, between classes, and with mRNAs? By using live-cell super-resolution imaging, Park et al. (2021) provided a dynamic view of interactions between the RNA chaperone Hfq, sRNAs, and mRNAs. Under normal growth conditions, the majority of Hfq is occupied by mRNAs during exponential growth, with the distal face contributing mostly over the rim to the mRNA binding. Class I sRNAs can co-occupy Hfq with both target and non-target mRNAs. On the contrary, Class II can effectively displace mRNAs from the distal face. This mRNA displacement by Class II sRNA requires both their interactions at the Hfq proximal face and the presence of a high A-rich motif content to outcompete mRNAs for binding at the distal face. Importantly, it was suggested that competitive binding of Class II sRNA to Hfq occurs in a stepwise manner, with binding at the proximal face occurring first, followed by displacement of mRNA from the distal face. Finally, Park et al. (2021) suggested that binding of Hfq to certain mRNAs through the distal face can recruit RNase E to promote the turnover of these mRNAs in an sRNA-independent manner. However, this regulatory function of Hfq can be challenged by Class II sRNAs by competing for binding to the distal face.

With the combination of these pieces of information, we can envisage an extraordinary multi-gate device (Figure 1) where: (i) sRNAs compete, regardless of Class, for the Hfq proximal face, (ii) mRNAs compete for the Hfq distal face, and (iii) Class II sRNAs, once they have gained access to the proximal face, compete with mRNAs for the distal face. The relative amounts (where Class I is more represented than Class II) and affinities of the sRNAs for the proximal and mRNAs for the distal face, respectively, determine the competition.

In this view, for an sRNA molecule to regulate through Hfq-assisted base-pairing, it must win the competition for the proximal face and be timely enough to find a target mRNA molecule bound to the distal face. Therefore, the competition among sRNAs for the Hfq proximal face can be an important gate of the base-pairing mechanism. In addition, a Class II RNA could further interfere with the base-pairing mechanism by reducing the likelihood of a target mRNA being bound to the distal face.

Furthermore, the mRNA displacement activity of Class II sRNAs may be an important player in modulating Hfq-mediated regulation of mRNA translation and stability. In an mRNA-specific manner, we can speculate that Class II sRNAs may be able to increase mRNA stability either directly, by challenging the recruitment of RNase E, or indirectly by alleviating the translational repression operated by Hfq, resulting in the protection of the mRNA from RNase E by translating ribosomes. Conversely, Class II sRNAs may also reduce the stability of certain mRNAs if Hfq binding plays the role of protecting them from degradation. It might be supposed that Class I sRNAs are excluded from this type of regulation. However, by competing with a Class II sRNA for the Hfq proximal face, Class I sRNAs may also regulate the translation and stability of non-target mRNAs.

In summary, according to this view of the Hfq hub, the sRNA-mediated regulation of mRNAs can be conveyed from the proximal to the distal face of Hfq with a mixed code of RNA–RNA and RNA-protein interactions. The decoy function of Class II sRNAs at the distal face would appear to be a key element in the modulation of Hfq mRNA binding activity and thus in the determination of gene regulation effects.

The small transcriptome also interacts with other RBPs, such as those in the CsrA/RsmA family (Quendera et al., 2020; Djapgne and Oglesby, 2021) of small homodimeric proteins, which are global regulators of translation, either positive or negative, through direct binding to mRNAs. Members of the CsrA/RsmA family recognize a core sequence of GGA (one per monomer) in the target RNA species, which is in the loop of a stem-loop structure. The mRNA binding activity of the CsrA/RsmA orthologs is mainly modulated by competition with sRNAs endowed with juxtaposed GGA motifs and thus able to titrate the RBP away from mRNA targets. For example, in *E. coli*, the activity of CsrA is mainly modulated by the sRNAs CsrB and CsrC, while *Pseudomonas aeruginosa* has two paralogs, RsmA and RsmN, and the four sRNAs RsmV, RsmW, RsmY, and RsmZ that function as antagonists. Thus, if the Hfq hub can be envisioned as multi-gated, the CsrA/RsmA RBPs support only one mechanism of competition, that between sRNAs and mRNAs for binding to the protein, which could be likened to what occurs at the distal face of Hfq. The antagonist sRNAs mentioned appear to be exclusively dedicated to the regulation of CrcA/RsmA orthologs by protein titration, and no base-pairing activity with mRNAs is known. However, sRNAs were discovered to perform both functions. In *E. coli*, McaS was characterized as an Hfq-dependent sRNA base-pairing to some targets (Thomason et al., 2012). McaS also contains two critical GGA binding motifs and can effectively remove CsrA from target RNAs (Jørgensen et al., 2013). More recently, the *E. coli* GadY, Spot 42, GcvB, and MicL, which are Hfq-dependent base-pairing sRNAs, were found to bind CrsA with high affinity (Potts et al., 2017; Lai et al., 2022). Like McaS, GadY contains GGA sites and was suggested to act at least in part by titrating CsrA (Parker et al., 2017). Interestingly, the interaction of CsrA with GadY, Spot 42, and GcvB was shown to significantly overlap with known regions base-pairing to target mRNAs (Potts et al., 2017). This suggests a competition between the protein-binding and base-pairing activities of these sRNAs, and again underscores the need to characterize many other aspects of the regulatory roles of the small transcriptome. In addition, there can be a regulatory effect of CsrA on sRNAs. For example, CsrA binding protects Spot 42 from RNase E-mediated degradation (Lai et al., 2022).

The above examples represent the set of rules, or codes, that govern the function of sRNAs as regulators of gene expression through base-pairing and protein-binding. Besides, the genetic code can also be used. Indeed, about ten sRNAs were shown to be dual-function, i.e., both regulators and mRNAs (Gimpel and Brantl, 2017; Raina et al., 2018). The small proteins encoded by dual-function sRNAs can act in the same physiological pathways as cognate riboregulators or in different pathways. This issue intersects with the emerging field of discovery and functional characterization of small proteins, commonly in the range of 50 amino acids (aa) or fewer in length (Ruiz-Orera and Albà, 2019; Orr et al., 2021; Steinberg and Koch, 2021; Gray et al., 2022). The role(s) of small proteins is still largely unknown (Steinberg and Koch, 2021). Small proteins may act primarily by stabilizing protein assemblies and/or modifying the activity of larger proteins. The production of small proteins is often associated with stress conditions, and thus their role could be as intracellular modifiers to adapt cellular physiology to different stimuli. The identification of new small proteins is expected to benefit from recent advances in mass spectrometry (Ahrens et al., 2022). In addition, powerful ribosome profiling techniques have also recently been described (Vazquez-Laslop et al., 2022), e.g., Ribo-RET which stands for Retapamulin-enhanced Ribo-seq analysis (Meydan et al., 2019). The antibiotic retapamulin specifically stops bacterial ribosomes at start codons, and Ribo-RET has the potential to detect known start sites as well as multiple new sites within or outside coding regions. In addition, ribosomal profiling studies in bacteria have suffered from relatively low resolution and have not provided reading frame information because of micrococcal nuclease used to degrade unprotected regions of mRNA. A method based on the RelE endonuclease has great potential for the improvement of the detection of translated open reading frames (Hwang and Buskirk, 2017). Indeed, RelE works only when bound to ribosomal site A and cleaves the mRNA after the second nucleotide in the site A codon.

Taking a recent ribosome profiling approach with stalled initiation complexes in *E. coli* as an example (Weaver et al., 2019), many new translation initiation sites of small proteins were identified. The corresponding ORFs were mapped in intergenic regions, antisense to other genes, in operons, and overlapping with other known ORFs. However, an estimate of how many sRNAs, for example in intergenic regions, contain these small ORFs and thus behave as smRNAs is not made explicit. Such an evaluation in this and future work would be very important to fill the gap that we have on the portion of sRNAs that are translated and thus use the genetic one as their functional code. It is quite predictable that approaches of this kind will greatly increase the number of sRNAs belonging to the translatome.

Furthermore, at least in *E. coli*, but it may be similar in other species, it is noteworthy that one-third of the small proteins are estimated to be localized in the cytoplasmic membrane (Yadavalli and Yuan, 2022). Although the details of bacterial mRNA targeting are still largely unknown (Irastortza-Olaziregi and Amster-Choder, 2021), recent evidence suggests that a rather unique localization pathway is used for small membrane proteins. This pathway is initiated by an mRNA targeting step that brings the mRNA to the membrane for subsequent translation by membrane-bound ribosomes, which account for ~10%–20% of all ribosomes in the cell (Herskovits et al., 2002). This could be the case for *E. coli* small membrane protein YohP and its smRNA (Steinberg et al., 2020). Future application of ribosome profiling protocols to membrane fractions may enable the identification of novel small membrane proteins and possibly also smRNAs.

## Discussion

**Figure 2:**
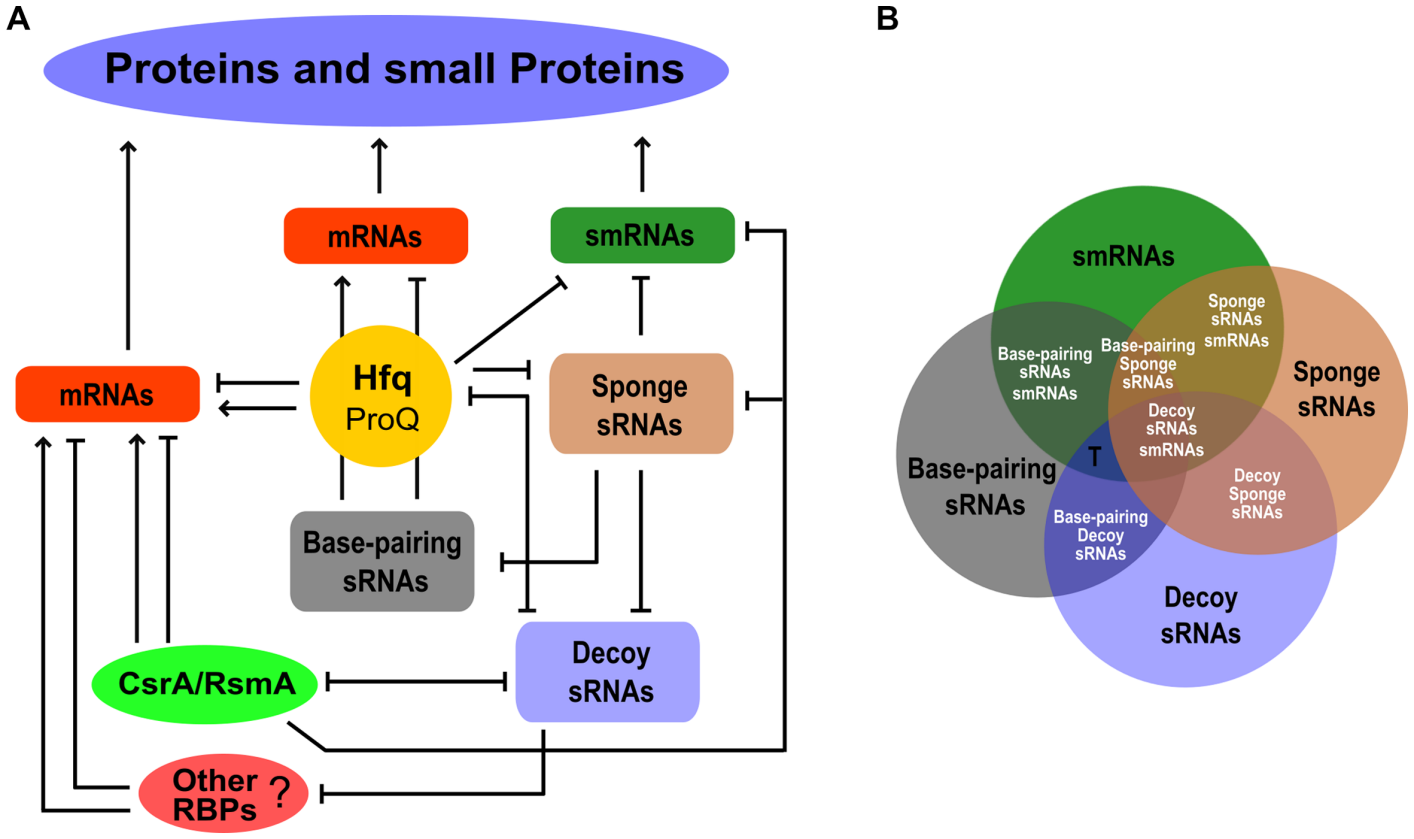
The functional network behind protein and small protein expression within the small transcriptome. **(A)** The different functional classes of sRNAs, i.e., base-pairing, decoy, sponge, and smRNAs, are shown as nodes of the network interplaying with each other or with RBPs. The post-transcriptional regulation of mRNAs by Hfq and possibly ProQ, CsrA/RsmA, and other unknown RBPs is also indicated. The smRNAs may also be the targets of these RBP-mediated regulatory effects. For simplicity, the Hfq hub shown in Figure 1 is not represented in this diagram. Arrows and lines ending with a short orthogonal dash indicated positive and negative effects, respectively. **(B)** Venn diagram showing the possible functional overlapping in an sRNA. Single-function and dual-function sRNAs are indicated by black and white fonts, respectively. The T letter indicates possible triple-function sRNAs as in the case of Spot 42.

Never more than in the last few years have we come to realize that the small transcriptome performs its function of regulation of gene expression through a combination of RNA–RNA and RNA-protein interactions. Moreover, some sRNAs can also be smRNAs. Figure 2A is just an attempt to illustrate this complexity driven by the different functional codes within the small transcriptome. Hfq is a highly trafficked and multifaceted node where various mRNA-mRNA, sRNA-sRNA, and sRNA-mRNA competitions for binding to Hfq, as well as sRNA-mRNA base-pairing, can take place. The same could occur with the FinO/ProQ proteins (Attaiech et al., 2017; Olejniczak and Storz, 2017). Decoy sRNAs can influence the activity of Hfq to regulate mRNA translatability and stability. The same effects of sRNA-mediated sequestration can involve other RBPs such as those in the CsrA/RsmA family. As in the case of Hfq, we can speculate a competition between decoy sRNAs for binding to CsrA/RsmA orthologs. Some decoy sRNAs can interact with both Hfq and CsrA/RsmA members. This could lead to an sRNA-mediated interplay between Hfq and the CsrA/RsmA proteins. In addition, if a decoy sRNA possesses base-pairing activity, competition may occur between it and protein-binding activity. Other unknown RBPs could participate in this game. Finally, by forming RNA duplexes, sponge sRNAs can inhibit all functional classes of sRNAs, i.e., base-pairing and decoy sRNAs as well as smRNAs. It is worth noting that many functional aspects of this scheme remain to be explored, and given the size of the small transcriptome, this poses a challenge for the future.

Recent global approaches to RNA biology (Hör et al., 2018) allow us to identify in the small transcriptome an increasing number of RNA–RNA and RNA-protein interactions and, in the latter case, to map precisely the RNA domains involved in the interaction. Recent advances in ribosome profiling, e.g., Ribo-RET or modified protocols with the drug lefamulin showing increased activity against Gram-negative species, should lead to the discovery of more smRNAs than the few we currently know about, i.e., to an enlargement of the small translatome. Since the expression and translation of sRNAs may be subject to physiological regulation, screenings under different growth conditions are expected to increase the likelihood of identifying new hits. Therefore, datasets of the various functional classes of the small transcriptome are expected to become increasingly populated. However, it is not enough to simply increase our collections of the small transcriptome, because this does not answer the questions raised above in the Introduction regarding the relative proportions of members belonging to the different functional classes and the number of multifunctional sRNAs.

To address these questions, a major future challenge will be to compare the different functional datasets to discover their degree of overlap (Figure 2B). From this perhaps we will find, for example, that many sRNAs have a unique function, e.g., only of smRNA, and that if we observe an association with Hfq or with a CsrA/RsmA protein, this is not to promote base-pairing with target mRNAs or for decoy, respectively, but to regulate the translation of the small encoded protein. Then, we may discover new dual-function sRNAs. Not only those base-pairing/smRNA and base-pairing/decoy of which some are already known, but also base-pairing/sponge, decoy/smRNA, sponge/smRNA, and decoy/sponge. In addition, the number of functions of an sRNA may not be limited to two and may even be triple. The case of Spot 42, one of the best-characterized small base-pairing RNAs in *E. coli* (Beisel et al., 2012), is very strongly illustrative of what may happen in the future by adopting this comparison strategy. The fact that Spot 42 contained an open reading frame (ORF) of 15 aa has been predicted since 1979 (Sahagan and Dahlberg, 1979). However, a 1987 study that examined the affinity between Spot 42 and the 70S ribosome and a fusion of the ORF of Spot 42 and *lacZ* led to the conclusion that Spot 42 did not function as an mRNA (Rice et al., 1987). In 2022, 35 years later, Gisela Storz and colleagues published that Spot 42 is a dual-function RNA that encodes a 15 aa protein that regulates the CRP transcription factor (Aoyama et al., 2022b). The suggestion for this strong breakthrough came from a Ribo-RET approach (Meydan et al., 2019; Weaver et al., 2019). Besides, as mentioned above, Spot 42 has been shown to bind with high affinity to CsrA and thus also has the potential to behave as a decoy sRNA (Potts et al., 2017). Therefore, we can say that Spot 42 received a nomination as a “triple-function” sRNA. This scenario of multifunctional sRNAs opens up further challenges, for example, understanding how the base-pairing function with target mRNAs affects smRNA function (Aoyama et al., 2022a), or how the decoy activity affects the base-pairing function when in the sRNA the domain for binding to the RBP coincides with the base-pairing domain.

In summary, in the small transcriptome resides an important share of code-driven information necessary for the adaptation of cellular physiology to environmental changes. Traditionally, sRNAs are generally considered to be noncoding, since only a few are known to contain translated ORFs. In this Perspective, we have tried to show that the number of sRNAs belonging to the translatome is likely to increase. In addition, the dynamics of the small transcriptome involve other functional codes and not only the genetic code. Therefore, we suggest a multi-code-driven functionality of sRNAs that would render the adjective noncoding misleading, as it only refers to “no coding for a protein.” Consequently, we believe that there is little reason to continue to call them noncoding sRNAs. Here, we have been focusing on sRNAs. However, given the generalized presence of multiple functional codes in RNA, sometimes coexisting on the same molecule, we like to think that in the future it will be avoided the use of the adjective noncoding even for RNAs such as rRNAs, tRNAs, miRNAs, and siRNAs, etc., which are very often belittled as such.

## Author contributions

SF, TB, and GB collaborated to conceptualize and write the manuscript. All authors contributed to the article and approved the submitted version.

## Funding

The research on sRNAs in the laboratory led by GB has been supported in recent years by the “Fondazione per la Ricerca sulla Fibrosi Cistica,” Verona, Italy, grant FFC#10/2020, with the contribution of Delegazione FFC di Firenze and Delegazione FFC di Prato, and grant FFC#14/2021 with the contributions of Delegazione FFC Ricerca Brindisi Torre, Delegazione FFC Ricerca di Prato, and Emanuela Cricri e Amici della Ricerca. The authors acknowledge support from the University of Milan through the APC initiative.

## Conflict of interest

The authors declare that the research was conducted in the absence of any commercial or financial relationships that could be construed as a potential conflict of interest.

## Publisher’s note

All claims expressed in this article are solely those of the authors and do not necessarily represent those of their affiliated organizations, or those of the publisher, the editors and the reviewers. Any product that may be evaluated in this article, or claim that may be made by its manufacturer, is not guaranteed or endorsed by the publisher.
